# Editorial: Efficient near-infrared-emitting materials: Design, synthesis, mechanisms, and applications

**DOI:** 10.3389/fchem.2022.1030420

**Published:** 2022-10-03

**Authors:** Dechao Yu, Lucas Carvalho Veloso Rodrigues, Chenghui Xia

**Affiliations:** ^1^ Engineering Research Center of Optical Instrument and System, The Ministry of Education, Shanghai Key Laboratory of Modern Optical Systems, University of Shanghai for Science and Technology, Shanghai, China; ^2^ State Key Laboratory of Luminescent Materials and Devices, South China University of Technology, Guangzhou, China; ^3^ Department of Fundamental Chemistry, Institute of Chemistry, University of São Paulo, São Paulo, Brazil; ^4^ School of Materials Science and Engineering, Ocean University of China, Qingdao, China

**Keywords:** lanthanide ions, transition metal ions, quantum cutting, quantum dots, phosphors

Near-infrared-emitting (NIR) materials have recently attracted attention in both academia and industry for the design of efficient NIR-active optoelectronic devices such as optical communications, light-emitting diodes, and photovoltaics, as well as for biomedical applications (Yu et al., 2022). With a wide collaboration and mutual complement of researchers in different fields, the development of efficient NIR materials with diverse compositions, shapes, and structures dramatically enriches the library of luminescent materials. Generally, NIR materials broadly contain lanthanide and/or transition metal ions-doped micro-/nano-phosphors and glass (ceramics), quantum dots, carbon dots, organic molecules, metal-organic frameworks, and some other organic-inorganic hybrid systems. Despite their great diversity, freely tuning excitation and emission for different purposes remains challenging. Moreover, the majority of NIR materials, both at the bulk level and on the nanoscale, typically have a poor luminescent quantum yield, lagging far behind the conventional luminescent materials with emissions in the visible region. To achieve efficient NIR emission, rational design and advanced synthesis, together with a fundamental insight into the emission mechanism, are worthy of comprehensive exploration (Yu et al., 2020). This exploration will also be beneficial for the realization of their full potential in various applications.

In this Research Topic, the reader will find some excellent works that shed new light on the design of NIR materials, modulation of their optoelectronic properties, discussion of their emission mechanisms, and demonstration of their practical applications. Gonçalves et al., at the Universidade de São Paulo, give a representative example of the design of super broadband rare-earth-doped SiO_2_-Ta_2_O_5_ glass ceramics and active planar waveguides at telecom wavelengths (De Oliveira Lima et al.). The emission bandwidth of the products can be rationally manipulated by tuning the concentrations and species of rare Earth dopants. Interestingly, triply (Er^3+^/Tm^3+^/Nd^3+^) doped SiO_2_-Ta_2_O_5_ nanocomposites exhibit pronounced NIR emissions, centered in the 1.5 µm region with a bandwidth of 173 nm, which is promising for photonic applications in optical devices operating in wide wavelengths at the telecom bands. Aside from rare-earth-doped materials, luminescent organic molecules with emissions in the NIR spectral range are also intensively explored. However, their quantum efficiencies are generally low since a small energy gap typically requires a planar molecular conformation which in turn prefers the formation of poorly emissive H-type aggregates (Spano and Silva, 2014). To combat this issue, Wang and co-authors report a regioisomerization strategy to convert aggregation-caused quenching molecules into aggregation-induced emission active organic molecules by simply migrating a small pyrrolidine group from *para*-to-*ortho*-position based on the rofecoxib scaffold (Wang et al., 2022). Surprisingly, the product molecules show a broad emission centered at 674 nm and their photoluminescence quantum yield significantly increases by at least a factor of 10. In addition, the compound exhibits mechanochromic luminescence behaviors which can be used as security ink. This piece of work is of great importance and provides a new guideline for the design of efficient luminescent organic molecules.

Though we intend to focus on presenting the state-of-art research findings on NIR materials, we also received some excellent works in the field of organic light-emitting diodes and biomedical applications, which may trigger the reader’s interest. Xu and co-authors systematically review the growth kinetics and function of an ultrathin emitting nanolayer technology which was recently developed in the fabrication of efficient organic light-emitting diodes (Xu et al.). The ultrathin emitting nanolayers, sandwiched between the exciplex interface of the hole transporting layer and the electron transport layer, can significantly enhance the efficiency of devices since these layers greatly improve carrier injection and exciton harvesting as well as achieve good exciton management (Xu et al., 2017; Zhang et al., 2020; Zhang et al., 2021). The group further investigates the underlying mechanisms of the ultrathin emitting nanolayers within interface exciplexes or nonexciplexes and discloses that the exciplex between 4,4′,4″-Tris (carbazol-9-yl)-triphenylamine and 4,7-Diphenyl-1,10-phenanthroline has a longer lifetime decay than the non-exciplex, thus facilitating exciton harvesting. This work reveals the function and mechanisms of ultrathin emitting layer technology, which is beneficial for the design of new types of highly efficient organic light-emitting devices. Another example is presented by Wang and co-authors who report a comprehensive study on the biosafety of Fe_3_O_4_/GO nanomaterials (Zhang et al.). The study shows that the cytotoxicity of Fe_3_O_4_/GO is time- and concentration-dependent. Moreover, exposure of Fe_3_O_4_/GO nanomaterials to living cells induces an increase in reactive oxygen species, calcium levels, and oxidative stress in mitochondria, thereby leading to cell apoptosis.

In summary, this Research Topic summarizes different types of novel NIR-emitting materials, highlights the difficulties that hinder the development of efficient NIR emitters, provides new perspectives on the solutions, motivates relevant researchers to understand fundamental photophysical problems, and demonstrates some practical applications of those materials. We deeply appreciate our contributors for their active participation in showing their recent findings in relevant fields. Though composed of a limited number of articles, we highly recommend the readers explore the details and we all believe you will benefit from this interesting Research Topic.
